# The clinical use of the platelet to lymphocyte ratio and lymphocyte to monocyte ratio as prognostic factors in renal cell carcinoma: a systematic review and meta-analysis

**DOI:** 10.18632/oncotarget.21108

**Published:** 2017-09-20

**Authors:** Xuemin Wang, Shiqiang Su, Yuanshan Guo

**Affiliations:** ^1^ Department of Urology, The First Hospital of Shijiazhuang, Shijiazhuang 050011, Hebei Province, China

**Keywords:** inflammation, platelet to lymphocyte ratio, lymphocyte to monocyte ratio, renal cell carcinoma, prognosis

## Abstract

Conflicting evidence exists regarding the effect of platelet to lymphocyte ratio (PLR) and lymphocyte to monocyte ratio (LMR) on the prognosis of renal cell carcinoma (RCC) patients. Here we quantify the prognostic impact of these biomarkers and assess their consistency in RCC. Eligible studies were retrieved from the PubMed, Embase and Web of Science databases. Pooled hazard ratios (HRs), odds ratios (ORs), and 95% confidence intervals (CIs) were calculated. Sixteen studies containing 6,223 patients met criteria for inclusion. Overall, elevated PLR was associated with poorer overall survival (OS, HR 1.76, 95% CI 1.41–2.19, *P* < 0.001), progression-free survival (PFS, HR 2.81, 95% CI 1.40-5.63, *P* = 0.004) and recurrence-free survival (RFS, HR 2.64, 95% CI 1.35–5.14, *P* = 0.004). Conversely, high LMR was correlated with more favorable OS (HR 0.62, 95% CI 0.51–0.77, *P* < 0.001) and RFS (HR 0.53, 95% CI 0.42–0.67, *P* < 0.001). Moreover, low LMR was significantly associated with some clinicopathological characteristics that are indicative of poor prognosis and disease aggressiveness. By these results, elevated PLR was associated with poor outcomes, while high LMR correlated with more favorable survival in RCC patients. Pretreatment PLR and LMR can serve as prognostic factors in RCC patients.

## INTRODUCTION

Renal cell carcinoma (RCC), the seventh most common cancer for male and the ninth for female worldwide, represents 2–3% of all malignances in adults [1]. According to estimates, there will be 66,800 new confirmed cases and 23,400 occurred deaths in China in 2015 [2]. Despite great progress in surgical procedures, immune-therapy and targeted treatment in managing renal mass, its long-term survival remains unsatisfactory largely because of common recurrence *in situ*, distant metastasis and poor response rate [3]. The identification of prognosis predictors may have clinical significance to instruct therapeutic decisions and follow-up arrangements. Although postoperative histopathological variables are presently the most widespread accepted factors for patients stratification [4], these parameters may not be thoroughly dependable. Additionally, since most prognosis predictors are assessed postoperatively, preoperative biomarkers are needed to early predict oncologic outcomes.

More and more evidence supports that inflammation exerts a crucial role in the pathogenesis and progression of various malignances, including RCC [5–7]. Systemic inflammatory response (SIR) is well-agreed to be reflected by many biochemical or hematological parameters. Circulating biomarkers, which stand for the condition of inflammation, are believed to be potential prognostic factors for RCC patients. Platelet to lymphocyte ratio (PLR), and lymphocyte to monocyte ratio (LMR), two inflammatory markers which are came from the blood cells, have gained prognostic value in in a number of malignant diseases, including RCC [8, 9]. These two biomarkers, are non-invasive and simple, which may be easily accessible prognosis predictors that could be applied to instruct clinical decisions. A number of researches have examined their role as prognosis predictors, nevertheless, the coherence and importance of the prognostic value of PLR and LMR are still needed to be explored.

Hence, a systematic review of the related literatures was performed to investigate the associations of pretreatment PLR and LMR in RCC patients with oncologic outcomes and to combine the results in a meta-analysis.

## RESULTS

### Search results

**Figure 1:**
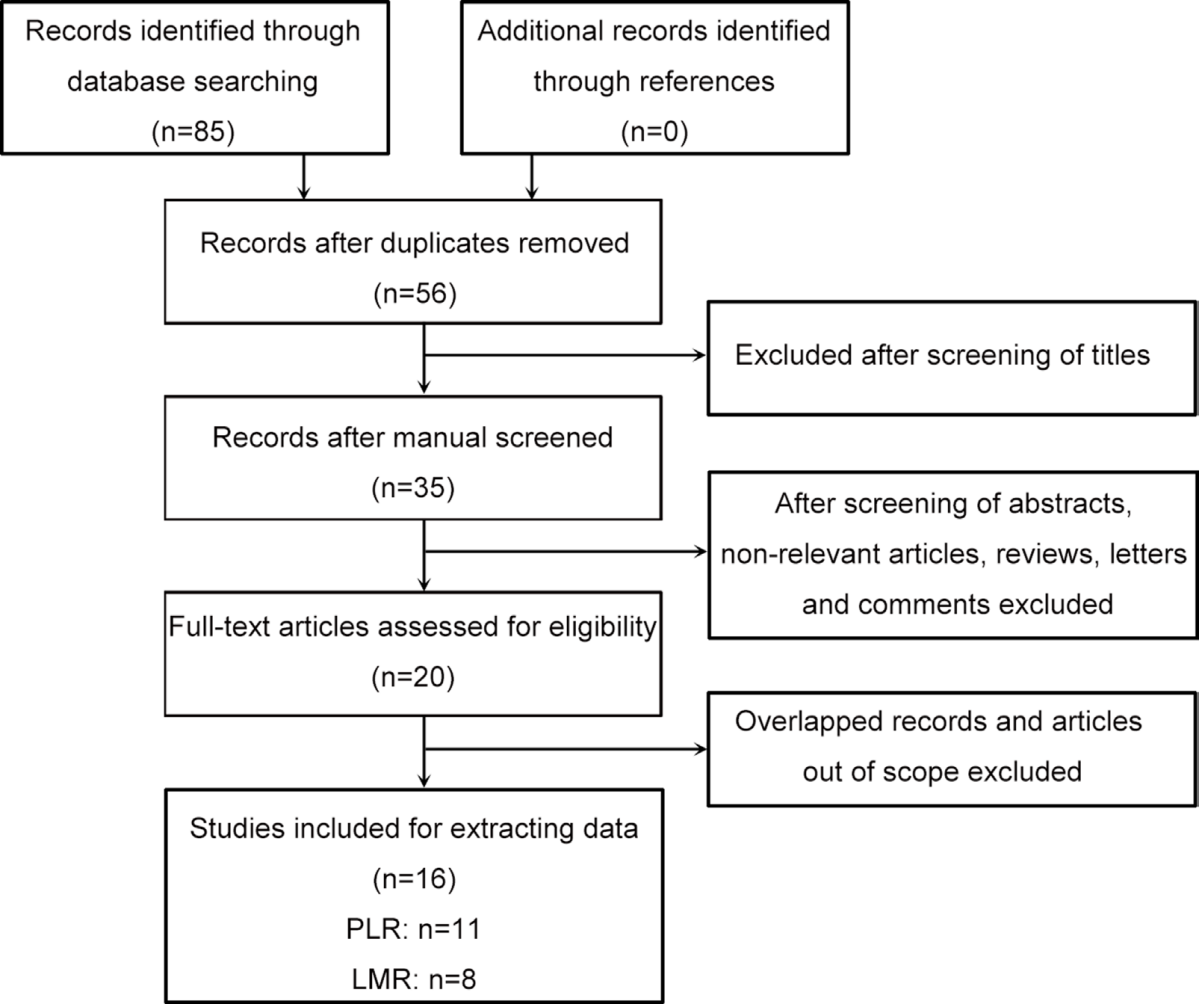
Flowchart shows the selection of literature for meta-analysis

A flow diagram of the study search process is presented in Figure 1. Totally, 85 records were identified through the primary study searching. Of all identified records, 29 were excluded due to duplicate studies. After screening the titles and abstracts, 20 literatures were remained. Then full-text screening was performed, 4 literatures were excluded because of overlapped records and out of scope. Finally, 16 literatures containing totally 6,223 patients, were included according to the eligibility criteria [10–25]. Among these literatures, 8 studied PLR, 5 investigated LMR, and 3 assessed both PLR and LMR.

### Characteristics of the studies

The baseline characteristics of the 16 literatures are outlined in Table 1. Most of the included studies had a retrospective design. All of them were published recently (2013–2017). Patients number ranged from 53 to 1360. The median or mean age of patients ranged from 55 to 65.5 years. Five literatures included all stages RCC, 7 studies only included metastatic RCC, and 4 studies only included non-metastatic RCC. Nine studies included all types RCC, 7 studies only included clear cell RCC. In 9 studies, the HR was adjusted for other covariates including histology, lymph node metastasis status, tumor stage, tumor site and size, Fuhrman grade, and tumor necrosis.

**Table 1:** Baseline characteristics of studies included in this meta-analysis

Study (year)	Country	Study design	Stage	Histology	Sample size	Median age	Cut-off value	Determine the cut-off value	Survival analysis	Source of HR	Adjusted	Follow-up (months)
Peng 2017	China	R	All	All	1360	55 (14–87)	PLR: 164/176 LMR: 4.3	ROC analysis	OS, PFS	SC	No	2–108
Ishihara 2017	Japan	R	M	All	63	–	PLR: 183	Maximum Youden index	OS, PFS	Rep	Yes	–60
Chrom 2017	Poland	R	M	All	321	62 (22–85)	PLR: 157	IMDC criteria	OS	Rep	Yes	–72
Xia 2016	China	R	NM	Clear cell	985	55 (21–81)	LMR: 3.0	25th percentile	OS, RFS	DE	No	3–60
Park 2016	Korea	R	M	Clear cell	63	63.1 (56–70.5)	PLR: 150	ROC analysis	OS, PFS	Rep	Yes	–
Hu 2016	China	R	All	All	484	56 (21–81)	PLR: 185	ROC analysis	OS	Rep	Yes	–60
Gu 2016	China	R	M	Clear cell	145	56 (47–63)	LMR: 3.0	ROC analysis	OS, PFS	Rep	Yes	–60
Gu 2016	China	R	All	All	103	56 (16–79)	PLR: 132 LMR: 3.1	ROC analysis	OS	Rep	Yes	–110
Lucca 2015	Austria	R	NM	Clear cell	430	65.5 (57–73)	PLR: 145 LMR: 2.5	Maximum survival difference	RFS	Rep	Yes	–48
Gunduz 2015	Turkey	R	M	All	94	58 (33–95)	PLR: 210	Maximum survival difference	OS, PFS	SC	No	–40
Chang 2015	China	R	NM	Clear cell	430	56 (46–63)	LMR: 3.25	25th percentile	RFS	Rep	Yes	–72
Chang 2015	China	R	All	Clear cell	441	56 (46–63)	LMR: 4.44	Median	OS	SC	No	–72
Keskin 2014	Turkey	R	All	All	211	61.2 (11.8)^a^	PLR: 151	Median	OS	SC	No	–24
Hutterer 2014	Austria	R	NM	Clear cell	678	65 (20–88)	LMR: 3.0	ROC analysis	OS, RFS	Rep	Yes	0–130
Fox 2013	Australia	P	M	All	362	62 (19–84)	PLR: 195	Median	OS	Rep	No	–40
Dirican 2013	Turkey	R	M	All	53	61 (40–79)	PLR: 134	Regression tree analysis	OS	DE	No	–40

Notes: study design: R retrospective, P prospective. Stage: All non-metastatic and metastatic, M metastatic, NM non-metastatic. Histology: All clear cell and non-clear cell. Survival analysis: OS overall survival, PFS progression-free survival, RFS recurrence-free survival. Source of HR: SC survival curve, Rep reported, DE data extrapolated.

-, not reported.

^a^ shown as mean (SD).

### Impact of PLR in OS, PFS and RFS of RCC patients

**Figure 2:**
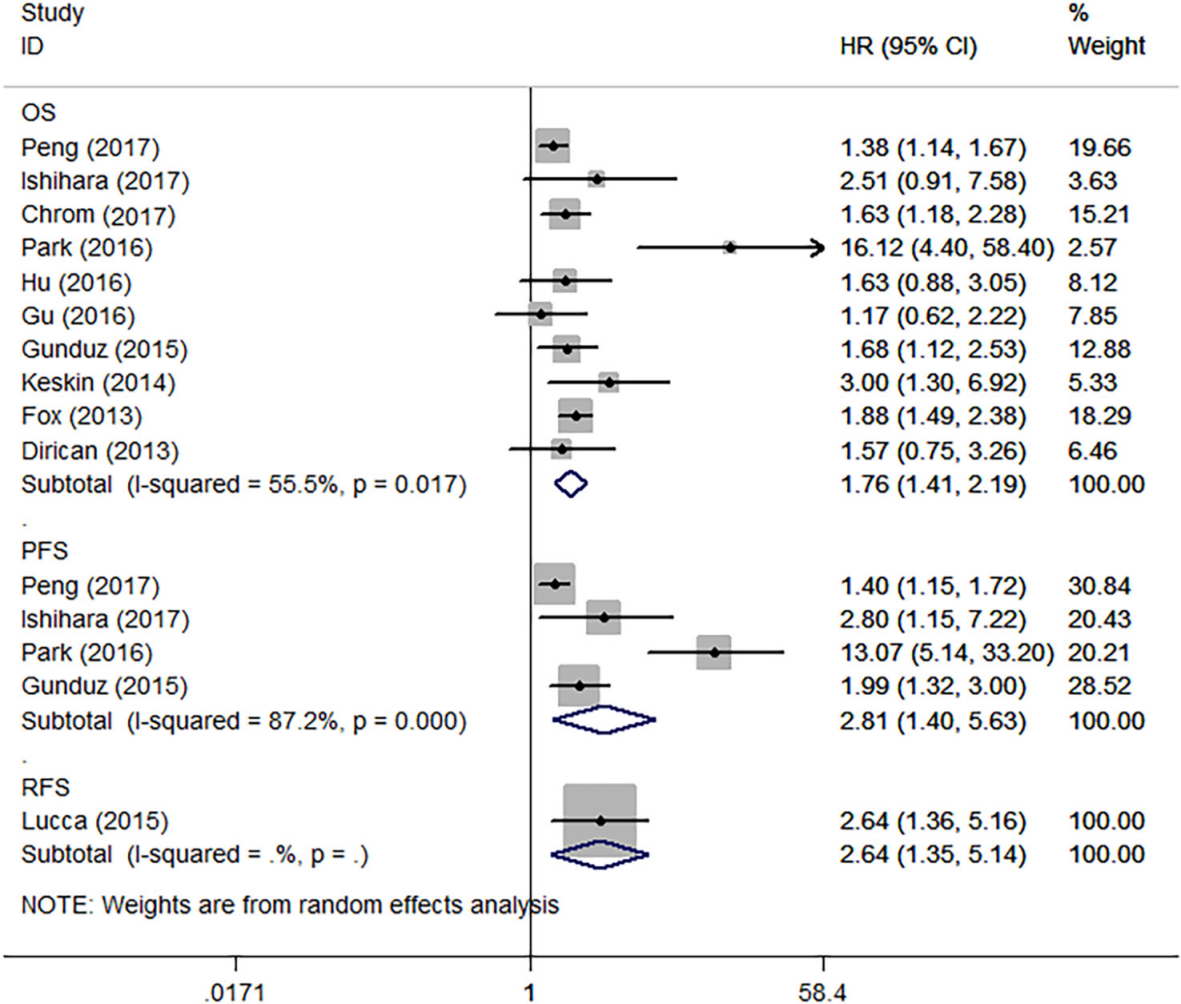
Forest plot reflects the association between PLR and oncologic outcomes OS = overall survival, PFS = progression-free survival, RFS = recurrence-free survival.

The association between PLR and oncologic outcomes was reported in 11 studies enrolling 3,660 patients [10–12, 14, 15, 17–19, 22, 24, 25]. Of these studies, 10 reported the results of overall survival (OS), 4 reported the results of progression-free survival (PFS), 1 reported the results of recurrence-free survival (RFS). Since significant heterogeneity exists among studies, a random-effect model was used in the analyses (I^2^ = 55.5% and 87.2%, *P* = 0.017 and < 0.001). As shown in Figure 2, after merging the data, we found that a high PLR was related to shorted OS (hazard ratio [HR] 1.76, 95% confidence interval [CI] 1.41–2.19, *P* < 0.001), and PFS (HR 2.81, 95% CI 1.40–5.63, *P* = 0.004). In addition, Lucca et al. [18] identified that patients with a high PLR experienced a shorter RFS (HR 2.64, 95% CI 1.35-5.14, *P* = 0.004). According to subgroup analysis, the HR of PLR on overall survival was 1.43 (95% CI 1.20–1.69, *P* < 0.001) for all stage (non-metastatic and metastatic) RCC, and 1.97 (95% CI 1.44–2.70, *P* < 0.001) for metastatic RCC patients. According to meta-regression analysis, year of publication, geographic region, cancer stage, sample size, cut-off value, source of HR, and ROC curve did not significantly contribute to inter-study heterogeneity (*P* = 0.178–0.786) (Table 2). According to sensitivity analysis, getting rid of any single literature did not significantly alter the pooled HR.

**Table 2:** Subgroup analysis of pooled hazard ratios for overall survival

Subgroup	HR (95% CI)	*P* value	Meta-regression *P* value	Heterogeneity	
				I^2^ (%)	*P* value
***PLR***					
Year of publication			0.284		
2016–2017	1.78 (1.23–2.57)	0.002		67.8	0.008
2013–2015	1.86 (1.53–2.25)	< 0.001		0.0	0.635
Region			0.295		
Asia	2.00 (1.15–3.47)	0.014		73.7	0.004
Non-Asia	1.80 (1.52–2.12)	< 0.001		0.0	0.709
Stage			0.178		
Mixed	1.43 (1.20–1.69)	< 0.001		19.4	0.293
Metastatic	1.97 (1.44–2.70)	< 0.001		58.5	0.034
Sample size			0.726		
> 200	1.60 (1.40–1.82)	< 0.001		37.2	0.174
< 200	2.17 (1.18–3.99)	0.012		70.1	0.010
Cut-off value			0.653		
> 160	1.59 (1.39–1.82)	< 0.001		18.5	0.297
< 160	2.22 (1.24–3.99)	0.007		72.8	0.007
ROC curve			0.240		
Considered	1.96 (1.04–3.66)	0.036		78.8	0.003
Not considered	1.81 (1.54–2.13)	< 0.001		0.0	0.774
Analysis of hazard ratio			0.786		
Univariate	1.60 (1.40–1.83)	< 0.001		37.6	0.171
Multivariable	2.12 (1.21–3.71)	0.009		70.4	0.009
***LMR***					
Year of publication			0.771		
2016–2017	0.68 (0.59–0.80)	< 0.001		44.7	0.143
2014–2015	0.58 (0.35–0.96)	0.032		60.3	0.112
Histology			0.099		
Mixed	0.75 (0.63–0.89)	0.001		0.0	0.657
Clear cell	0.54 (0.44–0.68)	< 0.001		12.3	0.331
Sample size			0.365		
> 600	0.70 (0.60–0.82)	< 0.001		31.4	0.233
< 600	0.54 (0.39–0.73)	< 0.001		39.7	0.190
Cut-off value			0.515		
> 3.0	0.66 (0.47–0.93)	0.017		52.6	0.121
= 3.0	0.58 (0.45–0.74)	< 0.001		20.1	0.286
ROC curve			0.082		
Considered	0.72 (0.62–0.84)	< 0.001		0.0	0.399
Not considered	0.48 (0.35–0.65)	< 0.001		0.0	0.665
Analysis of hazard ratio			0.779		
Univariate	0.64 (0.45–0.92)	0.016		64.9	0.091
Multivariable	0.61 (0.47–0.77)	< 0.001		37.5	0.187

HR, hazard ratio; CI, confidence interval. PLR, platelet to lymphocyte ratio. LMR, lymphocyte to monocyte ratio.

### Impact of LMR in OS, PFS and RFS of RCC patients

**Figure 3:**
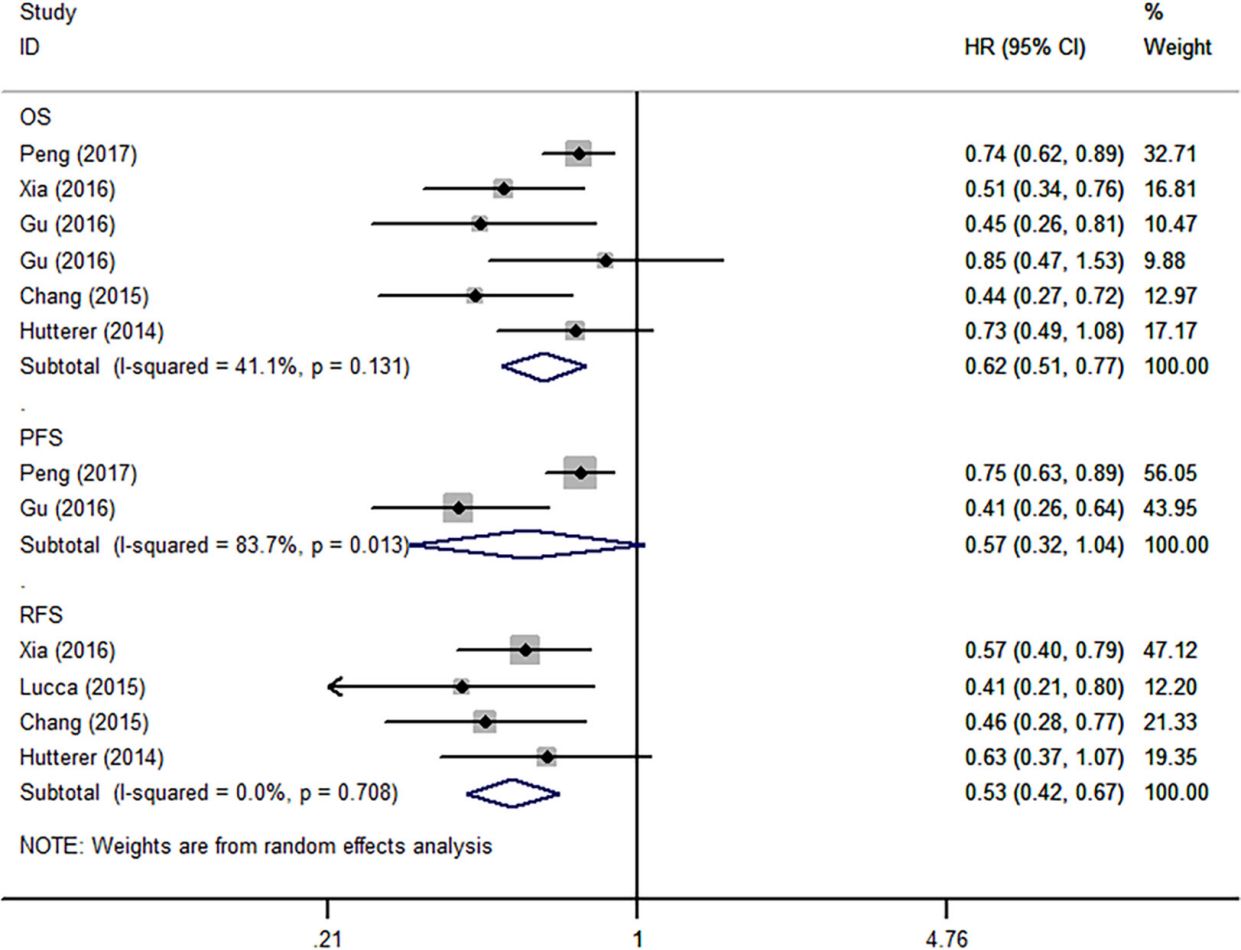
Forest plot reflects the association between LMR and oncologic outcomes OS = overall survival, PFS = progression-free survival, RFS = recurrence-free survival.

The relationship between LMR and oncologic outcomes was described in 8 literatures containing 4,572 patients [10, 13, 16–18, 20, 21, 23]. Of these studies, 6 reported the results of OS, 2 reported the results of PFS, 4 reported the results of RFS. As shown in Figure 3, after merging the data, we found that a high LMR was associated with superior OS (HR 0.62, 95% CI 0.51–0.77, *P* < 0.001) and RFS (HR 0.53, 95% CI 0.42–0.67, *P* < 0.001). However, the association between an elevated LMR and PFS (HR 0.57, 95% CI 0.32–1.04, *P* = 0.065) did not obtain significance. According to subgroup analysis, the HR of LMR on overall survival was 0.75 (95% CI 0.63–0.89, *P* = 0.001) for all types (clear cell and non-clear cell) RCC, and 0.54 (95% CI 0.44–0.68, *P* < 0.001) for clear cell RCC patients. According to meta-regression analysis, year of publication, histology, sample size, cut-off value, source of HR, and ROC curve did not significantly contribute to inter-study heterogeneity (*P* = 0.082–0.779) (Table 2). According to sensitivity analysis, getting rid of any single literature did not significantly alter the pooled HR.

### The relationship between LMR and clinicopathological characteristics

Four studies reported adequate data for the meta-analysis. As presented in Table 3, a high LMR was of a significant correlation with Fuhrman grade (III/IV vs I/II: odd ratio [OR] 0.63, 95% CI 0.53–0.76, *P* < 0.001), tumor necrosis (present vs absent: OR 0.66, 95% CI 0.54–0.79, *P* < 0.001), tumor size (> 7 vs < = 7: OR 0.59, 95% CI 0.51–0.69, *P* < 0.001), pT stage (pT3–4 vs pT1–2: OR 0.60, 95% CI 0.49–0.73, *P* < 0.001). Nevertheless, the correlation between a high LMR and lymph node status (positive vs negative: OR 0.60, 95% CI 0.18–2.00, *P* = 0.410), TNM staging (III/IV vs I/II: OR 0.61, 95% CI 0.26–1.41, *P* = 0.245) was not significant.

**Table 3:** Meta-analysis of the association between elevated LMR and clinicopathological features of renal cell carcinoma

Variables	Studies	Patients	Pooled OR	95% CI	*P* value	Heterogeneity *I*^2^ (%)	*P* value
Fuhrman grade	3	1264	0.63	0.53–0.76	< 0.001	0.0	0.463
Tumor necrosis	3	1264	0.66	0.54–0.79	< 0.001	7.4	0.340
Tumor size	2	1426	0.59	0.51–0.69	< 0.001	0.0	0.418
pT stage	3	1808	0.60	0.49–0.73	< 0.001	0.0	0.587
Lymph node status	2	1130	0.60	0.18–2.00	0.410	65.6	0.088
TNM staging	2	1426	0.61	0.26–1.41	0.245	89.7	0.002

LMR, lymphocyte to monocyte ratio. OR, odd ratio. CI, confidence interval.

### Publication bias

**Figure 4:**
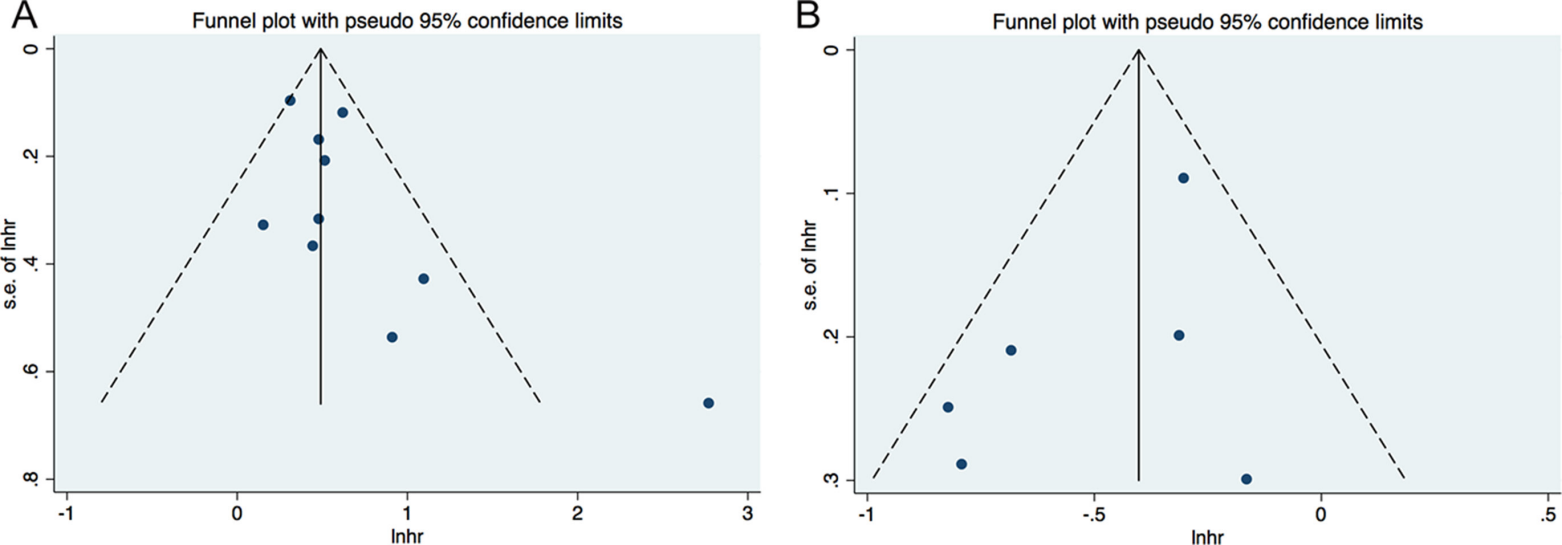
Funnel plot for publication bias (**A)** correlation of PLR with OS; (**B)** correlation of LMR with OS

In the literatures of correlations between PLR and LMR with overall survival in RCC, the funnel plots seem to be symmetry (Figure 4). The Egger's and Begg's tests were further performed. The results indicated absent evidence of significant publication bias for literatures about PLR and merged OS (Begg's test, *P* = 0.210; Egger's test, *P* = 0.095), and studies concerning LMR and pooled OS (Begg's test, *P* = 0.707; Egger's test, *P* = 0.221).

## DISCUSSION

Presently, the identification of prognostic biomarkers mainly focuses on tumor self- presentation and biological behavior, which might not stand for the authentic burden of RCC. The inclusion of the peripheral blood biomarkers as complementary items to existing prognosis prediction model is helpful in guiding clinical treatment strategy. Recent studies have identified that the markers of SIR are of prognostic significance in various cancer [8, 9, 26, 27]. Nevertheless, the coherence and importance of the prognostic value of PLR and LMR are still needed to be explored.

Hence, a systematic review of related literatures was conducted to assess the prognostic role of PLR and LMR in RCC. Then quantitative synthesis was performed using data from 16 studies embracing 6,223 patients. In the present study, we found that pretreatment PLR and LMR can be applied as prognostic factors for RCC cases. A high PLR was correlated with inferior OS, poor PFS and unfavorable RFS. To the contrary, a decreased LMR was associated with poor OS and RFS, but not for PFS. Moreover, subgroup analyses by year of publication, region, stage, histology, sample size, cut-off value, ROC curve, and source of HR did not affect the impact of PLR and LMR on OS. Our findings also showed that RCC cases having decreased LMR are prone to experience a higher nuclear grade, tumor necrosis, a larger primary tumor size, and a higher pathological T stage. Since there were no more than two studies report the same variables, we didn't merge the data about the associations between PLR and clinicopathological parameters. As PLR and LMR measurements are simple and easily accessible in each clinical centers, they can be useful and convenient circulating markers for decision-making.

The potential mechanisms of altered PLR and LMR affecting the oncologic outcomes of RCC patients remain speculative at this time. Elevated platelet count is commonly identified in patients with malignance, and is related to inferior oncologic outcomes [27]. Platelets may protect cancer cells from detecting or attacking by the autoimmune system. The potential mechanisms include platelets promote tumor cell adhesion to the vascular endothelium, or interact with cancer cells through its ligands [28]. Orellana et al. [29] cultivated human platelets and ovarian tumor cells together, and identified that interactions between platelet and cancer cell promoted metastasis formation. Moreover, obstruction of key platelet receptors hindered metastasis formation. Lymphopenia is an important component of elevated PLR. Lymphocytes stand for the cellular basis of immune-surveillance and immune-modifying, and lymphocyte penetration into the microenviroment of cancer act as a prior condition for the immune response against cancer [30, 31]. A low lymphocyte count might result in attenuation of immunologic antitumor reaction. However, monocytes infiltrating tumor microenviroment have a role in tumor development and progression [5]. Monocytes may be closely associated with the formation of tumor-associated macrophages (TAMs). More and more evidence supported that the TAMs enhance tumor progression. Based on these data, it is understandable that an elevated PLR and/or a decreased LMR lead to inferior survival.

Several underlying limitations of the present study should be admitted. First, though 16 literatures of 6,223 subjects were included in the present meta-analysis, only 11 articles studied PLR and 8 articles studied LMR. Due to the limited literatures, we analyzed metastatic and non-metastatic RCC together, which may introduce some inter-study heterogeneity. Second, obvious heterogeneity of studies was observed in several analyses. The inter-literatures heterogeneity was possibly because of differences in patients’ features (country, race, age, stage and histology), duration of follow-up, and the inconsistency of PLR and LMR cutoff values. Additionally, the type of HR and approach of calculating the hazard ratios also may result in heterogeneity. Of the 16 literatures, 10 reported HRs directly, and each HRs of the residual literatures were figured up with the methods described by Tierney et al. [32]. Among the 10 studies providing HRs, one reported univariate hazard ratio, which did not adjust for the potential confounding factors [24].

All included literatures were published in 2013 or later, indicating the present focus in studying the prognostic roles of PLR and LMR in RCC. Despite the above limitations, this systematic review and meta-analysis represents the most informative and comprehensive study evaluating this topic. Based on our findings, a high PLR was correlated with inferior outcomes, while elevated LMR was associated with relatively superior survival in RCC patients. The relative availability and low cost of these biomarkers should facilitate their use in predicting prognosis for RCC patients.

## MATERIALS AND METHODS

### Literature search

The databases of PubMed, Embase and Web of Science were methodically searched for article up to July 2017. The major searching terms included: “renal”, “cancer”, “platelet to lymphocyte ratio”, “lymphocyte to monocyte ratio” and “prognosis”. Additionally, we manually searched the bibliographies of relevant literatures for additional eligible studies.

### Study selection

The primary criteria weighed in including a literature were studying the oncologic outcomes of RCC, using pretreatment PLR or LMR as prognostic indicators and investigating their association with survival outcomes including OS, RFS and PFS. Articles were excluded if they (a) were presented in non-English; (b) were short of adequate data for calculating HRs and their 95% CIs; (c) reported PLR or LMR as continuous variables; (d) studied post-treatment PLR or LMR. When one center published more than one article about the same study population, we just included the most comprehensive and latest study. Two reviewers independently considered all the articles that met the inclusion criteria for full-text review. Any disagreements were discussed and arbitrated by a third reviewer.

### Data extraction and synthesis

According to the current interest, OS was the primary outcome, RFS and PFS were secondary outcomes. Two researchers independently collected necessary data. The data needs to be extracted as following: publication information (first author's last name, year of publication, geographic region, and study design), patients’ characteristics (sample size, age, follow-up time), cancer and outcomes (cancer stage, histology, oncologic outcomes, source of HR, adjusted or not), PLR or LMR data (cut-off value, the determining method). HRs of PLR or LMR for OS, RFS, PFS, as well as their 95% CIs were also extracted. If possible, HRs were obtained from multivariable analyses in prior. If not, HRs were obtained from univariate analyses. If the study did not provide HRs directly, we estimated individual HRs with the reported data (Kaplan-Meier curves or the necessary data) by applying the methods described by Tierney et al. [32] Subgroup analyses for OS were also performed to explore source of heterogeneity. The variables included year of publication, region, stage, histology, sample size, cut-off value, ROC curve, and source of HR.

We also studied the associations between LMR and clinicopathological parameters of RCC. Information about Fuhrman grade (III/IV vs I/II), tumor necrosis (present vs absent), tumor size (> 7 vs < = 7), pT stage (pT3–4 vs pT1–2), lymph node status (positive vs negative), TNM staging (III/IV vs I/II) were dichotomized. The event numbers were obtained from original studies, and the ORs and corresponding 95% CIs were calculated.

### Statistical analysis

Inter-study heterogeneity was measured by performing Cochran's *Q* test and Higgins I-squared statistic. There was marked heterogeneity if the *P* value was less than 0.10 and/or I^2^ > 50%. When the heterogeneity was identified, a random-effect model was applied. Otherwise, we used a fixed-effect model. A merged HR greater than 1 indicated a poor survival for the patients with a high PLR or LMR. The reasons for inter-study heterogeneity were also explored by using subgroup and meta-regression analysis. We evaluated publication bias with visual examination of funnel plots and precisely assessed by Egger's and Begg's tests when 6 and more literatures were embraced in the meta-analysis. Sensitivity analysis was also conducted by getting rid of any single literature to assess stability of the results. This study was conducted following the PRISMA guidelines and all data analysis was conducted with Stata 12.0 software (StatCorp, College Station, TX, USA).
